# Large gene overlaps in prokaryotic genomes: result of functional constraints or mispredictions?

**DOI:** 10.1186/1471-2164-9-335

**Published:** 2008-07-15

**Authors:** Albert Pallejà, Eoghan D Harrington, Peer Bork

**Affiliations:** 1 Biochemistry and Biotechnology Department, *Rovira i Virgili *University, C/Marcel·lí Domingo s/n, 43007 Tarragona, Catalunya, Spain; 2 European Molecular Biological Laboratory, Meyerhofstrasse, 1, 69012 Heidelberg, Germany; 3 Max Delbrück Centre for Molecular Medicine, Berlin-Buch, Robert-Rössle-Strasse 10, D-13092 Berlin, Germany

## Abstract

**Background:**

Across the fully sequenced microbial genomes there are thousands of examples of overlapping genes. Many of these are only a few nucleotides long and are thought to function by permitting the coordinated regulation of gene expression. However, there should also be selective pressure against long overlaps, as the existence of overlapping reading frames increases the risk of deleterious mutations. Here we examine the longest overlaps and assess whether they are the product of special functional constraints or of erroneous annotation.

**Results:**

We analysed the genes that overlap by 60 bps or more among 338 fully-sequenced prokaryotic genomes. The likely functional significance of an overlap was determined by comparing each of the genes to its respective orthologs. If a gene showed a significantly different length from its orthologs it was considered unlikely to be functional and therefore the result of an error either in sequencing or gene prediction. Focusing on 715 co-directional overlaps longer than 60 bps, we classified the erroneous ones into five categories: i) 5'-end extension of the downstream gene due to either a mispredicted start codon or a frameshift at 5'-end of the gene (409 overlaps), ii) fragmentation of a gene caused by a frameshift (163), iii) 3'-end extension of the upstream gene due to either a frameshift at 3'-end of a gene or point mutation at the stop codon (68), iv) Redundant gene predictions (4), v) 5' & 3'-end extension which is a combination of i) and iii) (71). We also studied 75 divergent overlaps that could be classified as misannotations of group i). Nevertheless we found some convergent long overlaps (54) that might be true overlaps, although an important part of convergent overlaps could be classified as group iii) (124).

**Conclusion:**

Among the 968 overlaps larger than 60 bps which we analysed, we did not find a single real one among the co-directional and divergent orientations and concluded that there had been an excessive number of misannotations. Only convergent orientation seems to permit some long overlaps, although convergent overlaps are also hampered by misannotations. We propose a simple rule to flag these erroneous gene length predictions to facilitate automatic annotation.

## Background

The exponentially increasing amount of sequence information has spurred the need for automated and accurate large-scale prediction and functional annotation of genes. A new generation of technologies is speeding up the sequencing even more, but this comes at the price of some biases and an increased error rate [1,2]. Thus, it is important to investigate unexplained phenomena for systematic errors. One such phenomenon is a large number of annotated genes with long overlaps. Overlapping genes are frequently observed in microbial chromosomes. Although they were initially found in the genomes of bacteriophages, animal viruses and mitochondria [3-5], they currently represent an important part of the genes in the fully sequenced prokaryotic genomes [6]. Furthermore, it is already known that overlapping pairs are conserved across species [7], and it is likely they have more homologs than genes that do not overlap. This makes the overlapping gene pairs highly valuable as a tool for function prediction as other structural prokaryotic features such as well-conserved operons, conserved distances between adjacent genes, COG groups or KEGG pathways have been used to infer functions in genomic and metagenomic data [8,9]. However, they still remain strongly affected by sequencing and annotating errors [10]. Among the fully sequenced microbial genomes, thousands of overlapping gene pairs have been predicted in all three transcriptional directional classes (co-directional (→→), convergent (→←) and divergent (←→) [5,11,12]. The overlaps can arise when the 3'-end of one of the genes in a pair is extended because a stop codon has been deleted, or because the stop codon has been disrupted by a point mutation or a frameshift mutation [7,11,13]. However, the overlaps can also arise through the elongation of the 5'-end of a gene because an alternative upstream start codon has been used [13-15]. While there is plenty of evidence that small gene overlaps of several nucleotides enhance coordinated transcription of functionally related genes [6-8,11,13,15], it is not known whether long overlaps are the product of special functional constraints or simply of large-scale misannotations. For bacterial genomes it has been reported that overlaps longer than 20 bps have a reduced Shine-Dalgarno (SD) prediction percentage [16]. This regulatory motif appears to work in concert with the start codons as part of an elaborate regulatory system for gene expression. Therefore, one possible explanation for this low percentage is that many of these genes are incorrectly annotated.

A number of previous studies of overlapping microbial genes suggested that annotation errors such as misprediction of start codons, loss of termination codons as well as the misidentification of the entire open reading frames (ORFs) can influence the statistics of overlapping genes and hence their analysis [6,7,11-15] (Table 1). These studies used to exclude from their analysis both the genes coding for hypothetical proteins and the genes whose start codons have been assigned differently by the annotation programs and have therefore been deposited with different coordinates in the databases. On the other hand, the authors tend to accept the gene pairs that are conserved in the COG database [17]. Only Rogozin *et al*. [14] have tried to find out how the overlapping genes evolve and have examined some long convergent overlaps. Nevertheless none of the previous studies has attempted to quantify and characterize rigorously these possible misannotations to be able to study gene overlaps more reliably. Here we analyse long overlaps between well-characterized genes to discriminate true events from misannotations and to use this knowledge to develop rules for improving gene annotation.

**Table 1:** Analysing previous overlapping genes reports

**Reference**	**Objective**	**Excluded genes**	**Accepted gene set**	**Annotation errors suggested**
**Fukuda et al., 1999 **[11]**Fukuda et al., 2003 **[7]	Comparison study of overlapping genes in two Mycoplasma genomes. Study of overlapping genes in bacterial genomes	Homologous genes whose start codons was assigned differently and genes coding for hypothetical or putative proteins	Authentic ORFs, thus genes not annotated as hypothetical or putative proteins and conserved in COG database	Misprediction of the start codons

**Rogozin et al., 2002 **[12]	Study of non-coding DNA in prokaryotic genomes	Genes coding for hypothetical proteins and overlapping more than 90 bps	Gene pairs not annotated as hypothetical or putative proteins and conserved in COG database	Misprediction of start codons, falsely predicted genes and missed genes, frameshifts

**Rogozin et al., 2002 **[14]	Analysis of the purifying and directional selection in overlapping prokaryotic genes	Genes not conserved in COG database and neither co-directional nor divergent overlapping pairs nor overlapping gene pairs not conserved in two or more species	Convergent overlapping genes conserved in both the COG database and in two or more than two genomes	Misprediction of start codons (affecting co-directional and divergent overlaps) and loss of termination codons (affecting co-directional and convergent overlaps)

**Johnson and Chisholm, 2004 **[6]	Study of the properties of the overlapping genes in microbial genomes	Genes coding for hypothetical proteins	Gene pairs not annotated as hypothetical or putative proteins	Misidentification of coding sequences

**Sakharkar et al., 2005 **[13]	Comparison study of overlapping genes in two Rickettsia genomes	Genes coding for hypothetical proteins	Gene pairs not annotated as hypothetical or unknown proteins	Incorrectly annotated ORFs

**Cock and Withworth, 2007 **[15]	Study of the relative reading frame bias in Prokaryotic Two-component system genes which use to overlap	Genes with ambiguous locations	Two component system gene pairs well located in the chromosome	Invalid bacterial start codons or premature stop codons

Comparison of previous overlapping genes studies. Columns referring to the authors, the authors' objectives, the genes excluded from their study, the genes accepted for their study, and the misannotations which they suggest are present in prokaryotic chromosomes.

## Results and Discussion

Usually, adjacent genes in prokaryotic chromosomes tend to be separated by a short intergenic distance or overlap by some base pairs in a preferred phase [6,12,14,15]. Particularly common are overlaps where the stop codon of the upstream gene is overlapping with the start codon of the downstream gene (overlaps of 1 or 4 bps) [6,7,11,14,15,18]. Overlapping genes among prokaryotes represented around 17% (173,663 overlapping pairs) out of the total gene pairs contained in 338 microbial genomes (1,016,129 gene pairs). Although it is lower percentage than some authors have reported before [6], those overlapping genes are a consistent feature of the prokaryotic chromosomes and are worthy of study. Of these 173,663 overlaps we selected 42,055 where both genes were well-characterized for our study. Among the prokaryotic overlaps, those with co-directional overlaps were clearly the most frequent, reflecting the fact that this is the most common orientation of two adjacent prokaryotic genes [18]. Furthermore, the genes in the prokaryotic chromosomes tend to be grouped into operons of functionally related genes and usually, those genes of a given operon are on the same strand [19-24]. In fact, co-directional overlaps represented around 92% (38,563 overlaps) of the well-characterized overlaps considered here, while convergent overlaps represented 7% (3,035) and divergent overlaps 1% (457). Of these overlaps, we chose a set of 968 overlaps longer than 60 bps that had consistent coordinates in three different databases.

### Types of misannotation

We were looking for functional overlaps among the 968 overlaps longer than 60 bps. Every gene of the overlapping pairs was compared with its orthologs. If there is a difference in gene length between the gene and its orthologs the overlap is probably unreal and caused by a sequencing or annotation error in one of the genes of the overlap. This difference in gene length could also mean that the overlap is real though unconserved and therefore, not functional. Although we can not definitively distinguish between these two facts, by categorizing the long overlaps manually, we can notice patterns that provide us with hints. For a list of all the overlaps manually analysed here see Additional file 1.

**Figure 1:**
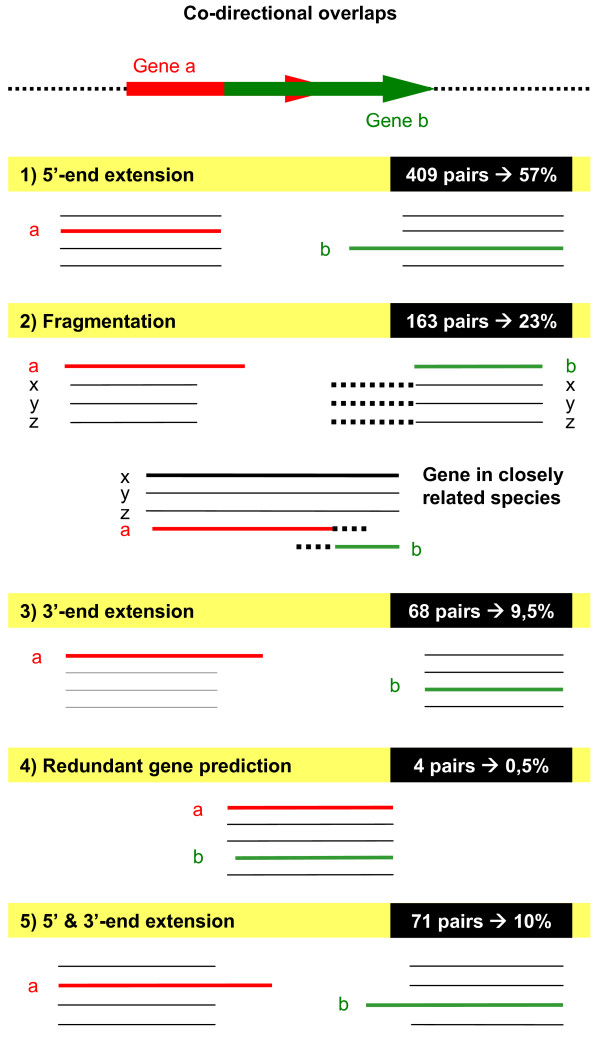
**Types of misannotation**. Schema of the five categories of putative misannotations. Both the number and the percentage of co-directional overlapping pairs longer than 60 bps classified in each group is shown. Gene a represents the upstream gene, while gene b represents the downstream gene. In Fragmentation type gene x, y and z represent the orthologs of gene a and b.

First of all, we manually analyzed 715 co-directional overlaps longer than 60 bps. Surprisingly all of them fell into the following categories (Figure 1):

i) 5'-end extension of the downstream gene due to either a mispredicted start codon or a frameshift at 5'-end of the gene. The upstream gene had the same length as its orthologs, while the downstream gene was longer than its orthologs at the 5'-end. Furthermore, in all the 409 cases classified, the downstream gene had alternative start codons which were downstream of the predicted initial codon, which could produce a product with a similar or even an equal length to its orthologs. These cases represented around 57% of the co-directional overlaps longer than 60 bps analysed. Therefore this suggests that the most important cause of long overlaps is a misprediction of the start codon of a gene;

ii) Fragmentation of a gene caused by a frameshift. In these cases the upstream gene was longer than its orthologs at the 3'-end and the downstream gene was clearly shorter than its orthologs. Furthermore, in these 163 cases both members of the overlapping pair could be mapped to a single gene in a closely related species, suggesting that a frameshift mutation/sequencing error fragmented one gene into an overlapping pair. These cases represented around 23% of the co-directional overlaps longer than 60 bps analysed and therefore, this is the second most important group of misannotations.

iii) 3'-end extension of the upstream gene due to either a frameshift at 3'-end of gene or point mutation at the stop codon. The upstream gene was longer than its orthologs at the 3'-end, whereas the downstream gene had a similar length to its orthologs. Either a frameshift at the 3'-end or a point mutation at the stop codon may cause the loss of the stop codon, thus extending the reading frame to the next in-frame stop codon. We found 68 cases (9,5% of the co-directional overlaps analysed) that showed this pattern.

iv) Redundant gene prediction where the genes overlap entirely or almost entirely and are in the same reading frame. This is a really strange case and actually we only found 4 gene pairs (0,5%), most of them labelled as putative genes.

v) 5' & 3'-end extension which is a combination of i) and iii). The upstream gene is longer than its orthologs at the 3'-end as well as the downstream gene being longer than its orthologs at the 5'-end. We classified in this group 71 overlaps (10%).

Regarding the overlapping lengths, the overlapping mean length of the 5', 3' and 5' & 3'-end extension groups was 104, 121 and 106 bps respectively. Nevertheless, the overlapping mean length of the fragmentation type was 162 bps, therefore this type of misannotations appears to cause longer overlaps. In order to know what type of misannotations causes the longest overlaps, we did not take into account the lengths of the overlaps caused by redundant gene prediction, because the gene pair is overlapping entirely or almost entirely and actually this type of misannotations occurs very rarely.

Although we extensively focused on the co-directional orientation, we also examined the long overlaps in the other orientations, specifically, 75 divergent overlaps and 178 convergent overlaps longer than 60 bps. All the divergent long overlaps belonged to group i), which means that all of them were misannotations due to a 5'-end extension of one or both genes of the divergent overlap. However, among the convergent overlaps we found putative true overlaps. Actually, as other authors have reported before [14], conserved convergent overlaps are affected by annotation errors to a lesser extent because they are not affected by the high rate of misannotated start codons. However, we could classify 124 convergent overlaps into group iii) as misannotations. Therefore, the misannotations are also affecting convergent overlaps, particularly those misannotations caused by a 3'-end extension in one or both genes of the pair. The other 54 convergent overlaps might be real, although most of them are only conserved in very close species.

Thus, we can now suggest ways to correct 914 gene pairs and clear the respective overlaps that are the result of misannotations. These overlaps caused by misannotations represent around the 2% of the overlaps of well characterized genes (42,055). Therefore, this is worth taking into account in the annotation processes.

### Misannotations in prokaryotic genomes

**Figure 2:**
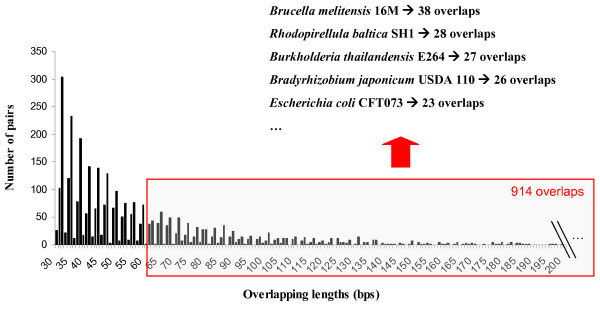
**Distribution of the overlapping pairs with respect to the overlapping length**. The longest overlaps selected for manual analysis are indicated by the red box. Several species contribute a disproportionate number of overlapping pairs to the misannotations. In the figure we can see the 5 species that accumulate more misannotations.

As expected, the number of overlaps decreases with an increasing overlap length (Figure 2). Equally expected is the avoidance of multiples of 3 bps overlaps for adjacent co-directional genes [6,14,15]. Although Figure 2 shows multiples of 3 bps convergent and divergent overlaps, none co-directional overlap was found with an overlapping length of multiple of 3 bps. We also studied in co-directional overlaps whether some particular genomes stood out in terms of overlaps because of their annotation protocols. Indeed, in some genomes large overlaps are more abundant with *Brucella melitensis *16 M leading with 38 likely misannotated events. Interestingly, 25 of those pairs were due to fragmentations [see Additional file 2]. Second in the list is *Rhodopirellula baltica *SH1, which has a really strange genome. It contains 28 misannotated overlaps, 26 of them are due to 5' or 5' & 3'-end extensions and it is the genome which has more divergent overlaps misannotated. Also we have observed that Xanthomonas genomes accumulated a high number of misannotations. Probably, the initial mispredictions in the first Xanthomonas genomes sequenced were propagated within this taxon due to the high sequence similarity among their genomes. For a list of 27 genomes with high number of overlaps see Additional file 3.

We tried to further identify reasons that might cause frameshifts and misannotations in the genome projects [see Additional file 3]. The genomes that accumulate a high number of errors are not the longest in size or the highest in gene content. For instance, the *Brucella melitensis *16 M chromosome has 3294931 nucleotides and 3198 predicted genes and accumulated 38 misannotations, whereas the *Vibrio vulnificus *YJ016 chromosome has 5211578 nucleotides and 5098 predicted genes but accumulated only 12 annotation errors. A high AT content could be related to a high number of mispredictions of start codons. However, no correlation between a high number of misannotations and a high percentage of AT was observed. We also did not observe any clear bias to any sequencing or annotation method, though 6 out of the 28 genomes worst annotated were done by Glimmer predictor [25] exclusively. However, the use of a determined gene predictor or a combination of different gene predictors, does not assure us that we will avoid the types of misannotations described here. The number of misannotations could also be related to the sequencing date. On one hand, an early sequencing date could be related to a high number of misannotations because less maturated technologies and tools were used. On the other hand, a recent sequencing date could be related to a high number of misannotations due to lower coverage and a higher degree of automation. However, no trend was observed in the number of misannotations regarding the sequencing date.

### Mispredicted start codons

5'-end extensions clearly have the highest number of misannotations because of mispredictions of start codons or upstream frameshifts whereby the former is clearly dominant (data not shown). Therefore we can say that the main problem in the annotation of real genes is the misprediction of start codons. Most genes tend to start with AUG while the alternatives GUG and UUG are used sparingly [16]. AUG is a more potent initiator than GUG or UUG [26], which are considered weak start codons. To quantify the observed effect regarding start codon usage, we compared the start codons of potentially misannotated genes with those from randomly chosen microbial genes. The genes which have putative mispredicted start codons (the genes with a 5'-end extension from wrong categories i), v) and from misannotated divergent overlaps group) had alternative start codons (AUG, GUG or UUG) downstream in the sequence. This could indicate that a gene with a mispredicted start codon has an additional correct one nearby. Furthermore, we observed statistical differences (P < 0.0001, Chi square analysis) which were extremely significant among the start codon usage between genes with a putative mispredicted start codon and a random set of genes. It seems that the use of the weak start codons (GUG, UUG) is overrepresented among the genes with putative mispredicted start codons [see Additional file 4]. We found that from the 579 genes, which potentially could have a mispredicted start codon, 270 start with AUG, whereas 172 and 133 with GUG and UUG respectively. In contrast, among the random sets of genes around ~462 start with AUG, whereas only around ~77 and ~38 with GUG and UUG respectively. Therefore, long overlaps, in conjunction with the use of weak start codons could be a sign that the 5'-end of an ORF has been mispredicted and must be taken into account by the annotation algorithms. In fact, some previous SD studies agreed with this finding. Starmer *et al*. explained genome annotation errors with a bias in the start codon prediction towards the usage of GUG instead of AUG [27], whereas a previous study performed by Ma *et al*. [16] found in *E. coli *K12 a significant group of genes which started with GUG or UUG and which do not have an SD sequence and hence were erroneously annotated as putative or hypothetical proteins.

### The longest real co-directional overlap

**Figure 3:**
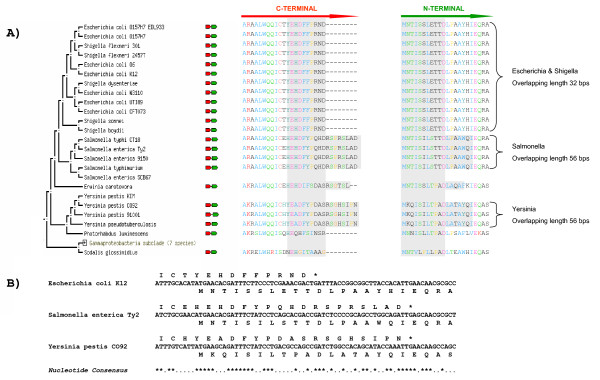
**Aligning a co-directional true overlap**. Overlap between the *holD *(coding for a DNA polymerase psi subunit) and *rimI *(coding for an alanine acetyltransferase) genes among Enterobacteria. A) Multiple alignment of the C-terminal of the DNA polymerase psi subunit and the N-terminal of the alanine acetyltransferase protein among Enterobacteria species. The grey boxes indicate the fragments that are encoded in the overlapping region between *holD *and *rimI *genes. The alignments of Escherichia & Shigella, Salmonella and Yersinia are marked. B) Arrangement of overlapping regions and amino acid conservation within the overlap among *Escherichia coli *K12, *Salmonella enterica *Ty2 and *Yersinia pestis *CO92. The nucleotide consensus shows an asterisk for the conserved nucleotides and a dot for the not conserved. Although we chose one species of each group marked in part A (Escherichia & Shigella, Salmonella and Yersinia) we can observe the high similarity at the level of sample nucleotide sequences too.

When studying co-directional overlaps below 60 bps, the longest real one we could identify was caused by two co-directional genes coding for the DNA polymerase psi subunit (*holD*) and an alanine acetyltransferase (*rimI*). Figure 3 shows the alignment of the C-terminal end of the DNA polymerase psi subunit and the N-terminal end of the alanine acetyltransferase as well as an arrangement of overlapping regions and amino acid conservation within the overlap among three representative Enterobacteria species. This figure highlights the high similarity among the Enterobacteria orthologs at the C-terminal end of the protein encoded in *holD *gene, at the N-terminal end of the protein encoded in *rimI *gene and within the overlapping region at the level of nucleotide sequence. This overlap was previously reported to be 32 bps long in *Escherichia coli *[28] which would correspond to around 10 overlapping amino acids; however orthologs gene pairs in the Yersinia and Salmonella genomes reached 56 bps, which would correspond to overlaps of about 18 amino acids. Although the exact gene length seems genus specific, this particular overlap is well conserved among Enterobacteria, and therefore unlikely to be due to a misannotation reported here.

## Conclusion

Misannotation of real genes leading to artificial extensions of genes seems to be more frequent than previously anticipated and can lead to frequent gene overlaps. We could show here that all co-directional and divergent overlaps extending 60 bps are artificial due to misannotations that can be classified into five categories. This clear-cut result enables us to propose a simple rule that can flag many thousand erroneous gene length predictions to facilitate automatic annotation. On the other hand, convergent orientation seems to allow longer overlaps than the other two orientations, although convergent long overlaps are also affected by misannotations.

The most common misannotation is the 5'-end extension, mostly caused by the misprediction of start codons. The respective genes carrying putative mispredictions of the start codon show an overrepresentation of weak start codons use. Thus genes with a 5'-end extension involved in long overlaps with predicted weak start codons must be checked by the annotation algorithms.

Although several species seemed to have a higher number of such potential misannotations, no correlation was found with genome size, gene content, GC content, sequencing or ORF prediction method, annotation team or sequencing date. Therefore these imprecise gene predictions have the potential to affect any microbial genome annotation process.

## Methods

Overlapping genes were retrieved from the 338 microbial genomes in the STRING database release 7.0 [29]. As has been mentioned above, analysis of the overlapping genes is hampered by sequencing and annotation errors present in genomes [10]. Because of this concern, only well-characterized genes were analysed. We defined as well-characterized genes only those gene pairs where both members could be assigned to a KEGG pathway [30]. This means that only 42,055 overlaps out of the 173,663 overlapping gene pairs observed among 338 prokaryotic genomes were considered in our study. Of these, 38,563 were in the co-directional orientation, whereas 3,035 were in convergent orientation and 457 were in divergent orientation. We focused on long overlaps to identify unusual differences in length. In order to avoid work with overlaps originated by inconsistent data among the databases, we checked whether their coordinates were consistent in STRING database release 7.0, Genome Reviews and RefSeq. We started analysing the longer overlaps and we stopped at 60 bps length because we observed conserved overlaps just below this cut-off.

After the application of all these restrictions commented on above, we eventually had 715 co-directional overlaps with overlapping lengths longer than 60 bps, which were examined manually. Each protein of these overlaps was compared to its corresponding orthologs, analogous to the consistency check used in the HAMAP project [31] for the SWISS-PROT protein validation. Therefore, for each member of an overlapping pair a multiple sequence alignment was constructed from the gene itself and its orthologs (as defined in the STRING [29] database) using Muscle [32]. These alignments were analysed by eye and if the overlapping genes showed significant differences in length, relative to their respective orthologs, we concluded that it was a misannotation. Then, these overlaps were placed into one of five categories based on putative sequencing or annotation errors that might have caused the artificial overlap. The convergent (178) and divergent (75) overlaps longer than 60 bps were also analysed manually. These overlaps were also placed into the categories previously defined with the exception of some of the convergent long overlaps.

We also examined whether certain species were associated with higher numbers of overlapping genes. In addition, we analyzed the correlation between the number of gene overlaps with genome size, gene content, GC content, sequencing or ORF prediction method, annotation team or sequencing date. We also analysed the misprediction of start codons using the genes that show 5'-end extensions among the groups 5'-end extension, 5' & 3'-end extension and the misannotated divergent overlaps, totalling 579 genes. The alternative start codons considered were AUG, GUG or UUG. The genes of genomes which use a different start codon to these three or a bacterial code different to the bacterial and plant plastic genetic code were classified as 'others' in the start codons table [see Additional file 4]. We checked the start codon in each case and how many times each of the three alternative start codons was used up to one third of the length of the gene. The figures were compared to normal gene sets randomly selected with two restrictions (random set I, II, and III). In the first one, the normal genes had to have gene lengths similar to the misannotation gene set (around 1400 bps). In the second one, the number of genes in each set had to be the same (that is, 579 genes in each set). We took well-characterized non-overlapping genes randomly selected as our normal genes. Furthermore, a Chi square analysis was performed comparing the start codon usage of one normal gene set with the mispredicted gene set. Where necessary we used Perl programming language in all the steps of this work as well as PostgreSQL to communicate with the STRING [29] database.

## Authors' contributions

AP performed the necessary Perl Scripts and sequence alignments and manually checked the overlaps. AP, EH and PB participated in the analysis and interpretation of the data. AP drafted the manuscripts and EH and PB revised it critically. Finally, all the authors read and approved the version to be published.

## Supplementary Material

Additional file 1 The 968 overlaps manually analysed. The co-directional, convergent and divergent overlaps analysed. They are separated depending on the orientation of the pair. The genes identification is made joining the Taxonomy ID of the species which contains the gene and the gene name separated by a dot. The columns are the upstream and the downstream gene ids, the functions of the protein encoded in the genes, the orientation, the overlapping length and the type of misannotation. Notice that the types of misannotations are described at the end of each of the lists. Click here for file

Additional file 2 Number of misannotations per genome in each category. Summary of the mispredicted overlaps found within the genome of each species sorted by categories. Click here for file

Additional file 3 Misannotations related to some genome features. Table summarizing the genomes with more misannotations and some features of the genome such as genome length, gene content, GC content, sequencing method, annotating method and sequence date. Click here for file

Additional file 4 Start codons analysis. Study of the start codons usage found among the three normal gene sets (random set I, II and II), which contains well-characterized non-overlapping genes randomly selected, and within the mispredicted start codon gene set. The usage and percentage of usage of each alternative start codon considered (AUG, GUG, UUG, other) is shown in the rows. Click here for file
