# Supplementary material for: Large gene overlaps in prokaryotic genomes: result of functional constraints or mispredictions?
Source: BMC Genomics. 2008 Jul 15;9:335. doi: 10.1186/1471-2164-9-335 (PMC2478687; doi:10.1186/1471-2164-9-335)
Supplement: Additional file 4 — Start codons analysis. Study of the start codons usage found among the three normal gene sets (random set I, II and II), which contains well-characterized non-overlapping genes randomly selected, and within the mispredicted start codon gene set. The usage and percentage of usage of each alternative start codon considered (AUG, GUG, UUG, other) is shown in the rows. [file 1471-2164-9-335-S4.doc]

|  | **mispredicted start codon** | **random set I** | **random set II** | **random set III** |
| --- | --- | --- | --- | --- |
| **number of genes** | 579 | 579 | 579 | 579 |
| **AUG usage** | **270** | **470** | **466** | **452** |
| **GUG usage** | **172** | **76** | **68** | **86** |
| **UUG usage** | **133** | **31** | **44** | **40** |
| **other start codons usage** | 4 | 2 | 1 | 1 |
| **% AUG usage** | 46,6 | 81,2 | 80,5 | 78,1 |
| **% GUG usage** | 29,7 | 13,1 | 11,7 | 14,8 |
| **% UUG usage** | 23,0 | 5,4 | 7,6 | 6,9 |
| **% other start codons usage** | 0,7 | 0,3 | 0,2 | 0,2 |
